# The Use of Remote Learning Techniques for Pediatric Cardiopulmonary Resuscitation Simulation Highlighting the Intersection Between Pediatric Advanced Life Support (PALS) and Advanced Cardiac Life Support (ACLS) Pathways

**DOI:** 10.7759/cureus.74974

**Published:** 2024-12-02

**Authors:** Stacey T Stokes, Lana Ismail, Kevin M Creamer

**Affiliations:** 1 Department of Pediatrics/Division of Hospital Medicine, Children's National Hospital, Washington, DC, USA

**Keywords:** pals, pediatric education, pediatric hospital medicine, simulation in medical education, tele-education

## Abstract

Introduction: Medical simulation education has expanded in the remote learning sphere, providing educational opportunities to under-resourced areas and the ability to engage learners limited by time or geographic location. Pediatric resuscitation training has historically been in-person relying on Pediatric Advanced Life Support (PALS) algorithms, yet many pediatric providers are often faced with treating adult or adult-sized patients. Our goal was to develop a tele-simulation remote learning module highlighting possible diagnoses and scenarios that require adult treatment-minded approaches for the pediatric clinician, including the use of Advanced Cardiac Life Support (ACLS) algorithms.

Methods: This simulation curriculum was offered in 2020 and 2022. The sessions were created for pediatric hospitalists and presented over an online video platform with visual aids to simulate being in a patient room. All three cases had the same base stem scenario and focused on a narcotic overdose, massive pulmonary embolism (PE), and torsades de pointes from polypharmacy-induced QTc prolongation. A multimodal assessment approach captured post-simulation comfort level with case content, information recall up to two years later, and pre- and post-test assessments of clinical knowledge improvement.

Results:Eighty-one simulation slots were filled by 56 clinicians over two years. Fifty-one completed course evaluations averaged a score of 4.95 on a 5-point Likert scale survey. Increased comfort level was statistically significant across all learning objectives. The most significant improvements were in the domains of ACLS algorithm use and understanding when to terminate unsuccessful resuscitation efforts. Pre- and post-test results demonstrated statistically significant (p<0.001) evidence of knowledge transfer in ACLS and PALS content.

Conclusion: Tele-simulation using an online video platform and well-defined visual aids is a useful option when in-person resuscitation simulation is not available. Practice and reinforcement of ACLS algorithms in pediatrics was found to be meaningful to clinicians who take care of patients of varying ages and sizes.

## Introduction

The case for medical simulation as an educational tool has previously been made by several studies that look at overall outcomes, necessary skill practice, and physician skill recall [1-14]. Much of this research and associated recommendations has been in the setting of in-person, hands-on simulation, through uninterrupted case simulation, rapid cycle deliberate practice, or a combination of the two [1-4]. During the COVID-19 pandemic, remote learning intensified and extended into prior underutilized areas, including simulation education. Historically, rural and under-resourced programs documented successful implementation of remote simulation [5-7]. More recently, there has been increasingly positive data relating to computer simulation with the neonatal resuscitation computer program RETAIN (REsuscitation TrAINing) and evolving data on remote surgery simulation [8-10]. During the COVID-19 pandemic, content related to remote simulation practices has shown promising outcomes [11,12], and standardized content such as Objective Structured Clinical Examinations (OSCEs) adapted to a remote learning and evaluation model with early encouraging results [13,14].

Pediatric physician resuscitation training heavily relies on Pediatric Advanced Life Support (PALS) algorithms; however, real-life situations often blur the lines between Advanced Cardiac Life Support (ACLS) and PALS pathways. Pediatric physicians commonly care for patients up to and including 21 years of age. Meanwhile with the rising obesity epidemic, weight-based dosing does not lend itself to pragmatic use, and obesity-related outcomes are becoming more common in pediatrics [15-17]. Few discussions are had about when to transition to ACLS resuscitation algorithms or what additional ACLS pathways should be considered in older pediatric patients.

The primary goal of this simulation was to use a tele-simulation remote platform to present three simulation cases with the same basic patient stem of an obese adolescent female patient and highlight three possible diagnoses and scenarios that require adult treatment-minded approaches, including ACLS methodology. Outcome measures of comfort level and clinical knowledge-based improvement were captured to evaluate success.

## Materials and methods

Development: Curriculum

Original resuscitation content within our Pediatric Hospital Medicine division was quarterly and in-person, focused on the hands-on skills required for advanced cardiopulmonary resuscitation featured in PALS. Sessions were 2-3 hours and offered several times each quarter. Early in the COVID-19 pandemic, the concern that pediatric physicians may need to care for sick adults inspired this round of remote virtual patient simulations focused on the intersection of ACLS and PALS to refresh skills and diagnostic heuristics that are more commonly encountered in the older pediatric and adult patient populations. This, along with the goal to not lose vital skills within the division, drove the simulation team to pivot to remote simulation.

Three simulation cases were created, all with the same base stem scenario, and pauses for educational discussions were scattered throughout. A virtual patient was designed using Adobe Photoshop™ (Adobe Inc., San Jose, CA) and Autodesk Maya™ (Autodesk, Inc., San Francisco, CA), and throughout each case, images were shown to provide participants with visual cues. The scenarios highlighted narcotic overdose, massive pulmonary embolism (PE), and torsades de pointes related to polypharmacy-induced QTc prolongation. Learning objectives were threefold: become more comfortable performing a rapid cardiopulmonary assessment on an adolescent patient with cardiopulmonary compromise, use the H’s and T’s mnemonic to help identify and treat causes of cardiopulmonary compromise and/or arrest, and utilize the appropriate pediatric and adult resuscitation pathways.

Development: Learner groups

Pediatric Hospital Medicine physicians require certification in PALS to be credentialed and practice at our institution. Between PALS recertification every two years, hospitalists are encouraged to voluntarily sign up for quarterly simulations within the division. Attendance is incentivized through CME and faculty development goals. Physician participants self-enrolled in the course through an online sign-up platform. An online video platform link was emailed close to the start date. Up to six hospitalists enrolled in each session to allow maximum participation in a small group setting.

Development: Personnel

The simulation facilitator created the cases, drawing on their prior critical care experience and literature [18,19] to inform the medical accuracy of each scenario. Scripted summaries for each of the cases guided the progression for a simulation technician to modify vital signs in real time. The simulation facilitator and technician reviewed these scenario summaries together ahead of time to allow seamless implementation and during the simulation sessions could private chat on the video platform to make small adjustments as needed. After each session, the simulation facilitator and technician would debrief and make small adjustments to improve the flow of the simulation prior to the subsequent sessions.

Equipment/Environment

All sessions of this simulation module were held using an online video platform (Zoom™, Zoom Communications, Inc., San Jose, CA) over a two-hour period. Prior to session start, the facilitator and technician set up co-hosting abilities to screen-share content to the group by toggling between the two of them. After the facilitator introduced the scenario, using visual aids in a presentation-style format within PowerPoint (Microsoft Corp., Redmond, WA), participants started their interventions. When they asked to place the patient on a monitor, the simulation technician showed vital signs in real time via a virtual Laerdal patient monitor (Laerdal, Stavanger, Norway). Once resuscitation commenced, the facilitator, using the private chat function, sent messages to the technician to change the displayed vital signs. While most of the expected interventions were programmed into the scenario summary, on-the-fly adjustments could be made for unexpected actions, using this communication system. Toggling between vital signs and patient images took place as needed or requested by participants. Supplemental images and didactic slides were at the end of each case to enhance teaching.

Piloting

Each quarterly simulation run by the simulation team always begins with a pilot session, where a smaller group of participants runs through the simulation, and volunteers critical feedback both verbally at the end of the session and in the anonymous post-simulation survey. This is also used to hone the timing of each case and confirm the overall time needed for the simulation session, which for this simulation was two hours. Enough time is allowed between that session and the next so that any changes can be made based on the feedback.

Examples of changes made to this curriculum were in the context of the PE case, where the patient outcome is death. In the pilot, it became clear that the outcome had a profound impact on providers, and the case was modified in three ways. First, an additional, optional debrief session was added several weeks later. It gave participants space to process their emotions regarding how the case impacted providers in the context of their clinical experiences. Second, the case itself was modified to add the ability to consult anesthesia and intensive care physicians during the decision-making process in order to add support to the decision to cease resuscitative efforts. Third, the case, which was initially the last case in the session, was moved to the second case for the session to not end with a patient death.

Implementation

This simulation was offered to participants twice, in the summers of 2020 and 2022, for reinforced learning and to capture new participants. The summer of 2022 simulation participants were separated into two groups, those who had participated in 2020 (“repeat” participants) and those who had no experience with the content (“new” participants) to avoid impacting the learning of new participants. With the 2022 sessions, additional quantitative and qualitative data was captured by the simulation team to assess the effectiveness and usefulness of the session.

The overall structure of the session began with an orientation didactic discussing PALS and ACLS specifics and highlighting differences with supporting PowerPoint slides, taking approximately 10 minutes. This was followed by cases with visual aids displaying the patient in their room, as well as physical examination findings via the same PowerPoint slide deck. Before the first case, visual aid slides were shown, and participants were read a scenario stem. The facilitator and simulation technician then followed the progression of the case. Appropriate PowerPoint slides were shown, and monitor changes were made based on the participants’ interventions in accordance with the scenario summary. As participants verbally provided interventions, it would prompt vital sign changes or probing questions. The facilitator would pause the case as needed to review findings in depth, answer questions, or go straight through the case prompted by students’ interventions and queries. There was an opportunity to discuss key learning points through supplemental slides (hidden slides in the slide deck) at the end of each case.

Group size and dynamics guided the simulation leader on the best approach to participation. While each session had participants paired to lead at least one of the cases, small groups often lent themselves naturally to group vocal participation in an orderly fashion by default, while larger groups or groups with more vocal participants worked well with the use of the chat box for non-leads to comment or ask questions. If one of the two co-leads was more vocal than the other, the facilitator would prompt the other lead for their opinion and the next action. Leader expertise in simulation education was used to determine which approach would work best with each class.

There were three cases presented to learners. All three cases, each covered once, featured a 17-year-old obese female patient with multiple comorbidities, a central line, and various medications who had a prolonged period of immobility due to extended hospitalization. The cases began with the provider entering to find her unresponsive. The first case required participants to use either the PALS bradycardia algorithm or the American Heart Association (AHA) Opioid-Associated Emergency for Healthcare Providers Algorithm. Basic concepts of acute opioid overdose management were discussed in a debrief of this session, including early naloxone consideration when opioid intoxication is suspected. Naloxone dosing for adults was highlighted.

The second case required either the PALS or ACLS Asystole/PEA algorithm, and the participants considered the “H’s and T’s” mnemonic to narrow diagnostic possibilities to massive PE. Ultimately, this case ended in the patient’s death despite all interventions. This case offered the opportunity to discuss risk factors for thrombosis, which are more common in older patients, and was followed by an extensive debriefing of how to approach stopping resuscitation efforts along with the medical and emotional elements using the CEASE (Clinical features, Effectiveness, Ask, Stop, Explain) framework [20].

The last case involved an unresponsive patient with torsades de pointes after QTc prolongation due to polypharmacy. Participants were expected to defibrillate per either the PALS or ACLS Ventricular Fibrillation/Ventricular Tachycardia (VFib/VTach) algorithm and review the “H’s and T’s”, resulting in return of spontaneous circulation (sinus rhythm) after an anti-arrhythmic medication is given. The importance of critically assessing medication side effects for all patients was highlighted in the debriefing of this case.

All cases touched on the intersection of PALS and ACLS in this late teenager with diagnoses more commonly seen in the adult hospital setting. Debriefing took place immediately after each case, consisting of a 5- to 15-minute unscripted discussion led by the facilitator. After assessing participants’ reactions, the facilitator solicited a brief one-line summary of the arrest scenario. Participants were allowed to analyze their performance with a modified plus/delta technique. They were afforded an opportunity to ask questions about specific aspects of the resuscitation scenario, and the facilitator highlighted important pearls that may have been missed during the simulation. Each participant was then asked what they would take away from the case prior to concluding and regrouping for the next case.

Assessment

Participants in 2020, after completing the simulation, were sent an anonymous Likert scale evaluation (1 = strongly disagree, 5 = strongly agree) with questions centered around participants’ comfort with steps of the cardiopulmonary assessment, using the “H’s and T’s” mnemonic, and managing torsades de pointes, among others. The simulation team reoffered the simulation in the summer of 2022 and captured data through the same Likert scale evaluation. The formula used to calculate the percentage in improved comfort is below, with improvement indicated by a before-simulation numerical value lower than the after-simulation numerical value: ((total # of respondents reporting improvement) / total # of respondents) X 100 = % respondents that reported improvement.

Also, in 2022, more robust data was captured to evaluate the effectiveness and appropriateness of the remote virtual patient simulation content through two additional layers of evaluation. First, to evaluate both learner information retention and content relevance, participants were sent a REDCap survey either two months after participating in the simulation (new participants) or 24 months after participation (repeat participants). The survey inquired about the use of the knowledge and skills learned during the simulation in their clinical practice and assessed their recall of the concepts in the simulation’s learning objectives. Second, all participants were sent a 10-question pre- and post-test, with five questions specific to the simulation cases and the remaining five covering general resuscitation content including Neonatal Resuscitation Program (NRP) and PALS materials to avoid clueing the participants into the content of our presentation. Test question writing used the learning objectives as a guide, with methodology from the National Board of Medical Examiners, and an iterative process for question creation and refinement among the group. We analyzed only the responses to the five questions specific to the simulation cases. Scores were evaluated for improvement using paired T-test statistical analysis.

## Results

In total, there were 81 simulation session slots filled by 56 participants (25 participated both in 2020 and 2022). In 2020, 43 Pediatric Hospital Medicine physicians participated in one of nine sessions, and in 2022, 38 physicians participated in one of 11 sessions, with an average of 3-5 physicians per session. We never ran a session with just one physician as we felt that solo participation would be too stressful. Teams of two would volunteer to take the lead before the beginning of each case. Depending on the number of participants in each session, everyone co-led at least one of the cases, while others were encouraged to participate via the chat function with ideas or comments.

Three modalities for course evaluation were completed (Table 1). Fifty-one (63%) out of 81 possible Likert scale (1 = strongly disagree, 5 = strongly agree) surveys were completed immediately after the simulation sessions. Participants rated the overall session with a mean score of 4.95 (Table 2).

**Table 1:** Participant Evaluation Modalities

Evaluation	Total completed
Likert scale surveys of content level of comfort and overall course evaluation	51
Supplemental follow-up survey	15
Pre- and post-knowledge test	26

**Table 2:** ACLS and PALS Simulation Attendee Course Evaluation ACLS: Advanced Cardiac Life Support, PALS: Pediatric Advanced Life Support

Likert scale statement (strongly agree = 5, strongly disagree = 1) (N=51)	Mean	Standard deviation	Median
My orientation to the virtual simulation event was adequate.	4.86	0.34	5
The cases were intellectually stimulating and engendered useful conversations.	4.98	0.14	5
The debriefing facilitator(s) was effective at conveying concepts and provoking thought.	4.96	0.19	5
The virtual simulation was a valuable use of time.	4.98	0.14	5

**Figure 1:**
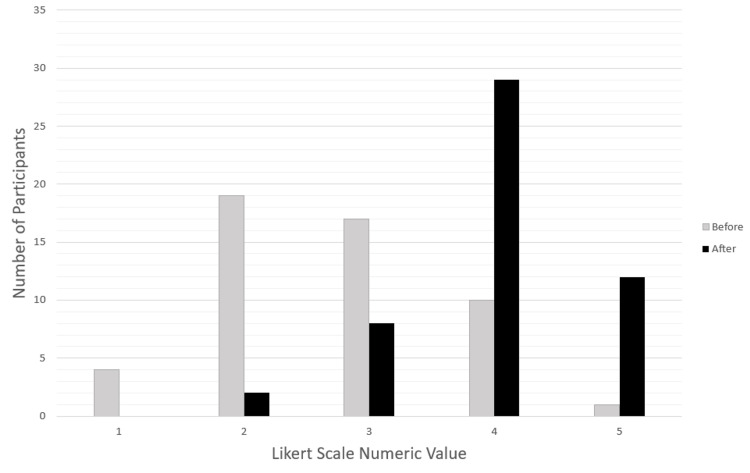
Likert Scale Responses (n=51) Before and After Training: I Am Comfortable Using ACLS Algorithms Likert scale: strongly disagree = 1, strongly agree = 5 ACLS: Advanced Cardiac Life Support

**Figure 2:**
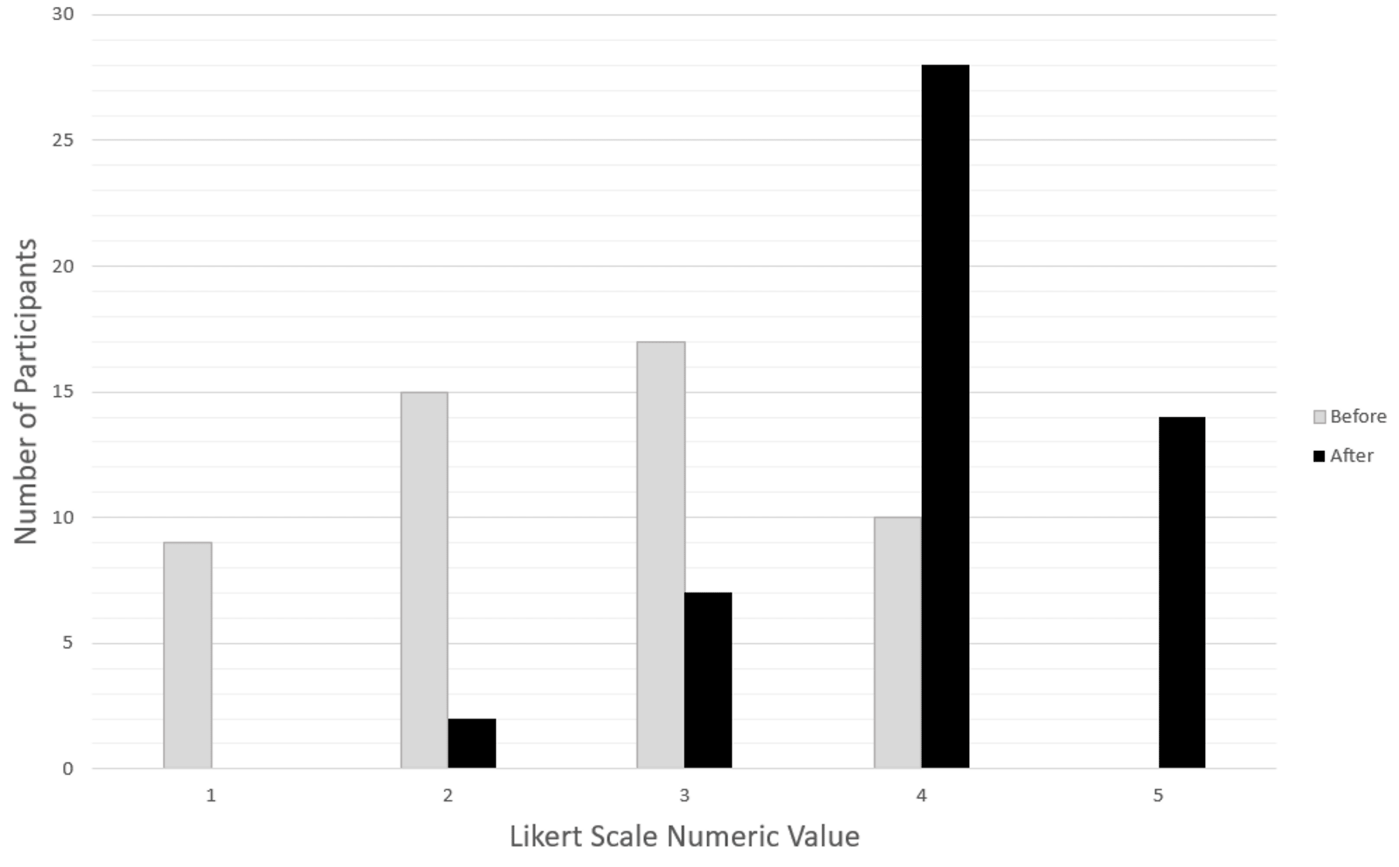
Likert Scale Responses (n=51) Before and After Training: I Am Comfortable Discussing When to Terminate Resuscitative Efforts Likert scale: strongly disagree = 1, strongly agree = 5

Survey before and after data demonstrated consistent statistically significant improvement in comfort levels of several diagnostic, algorithmic, and intervention-based parameters related to pediatric and adult resuscitation (Table 3). Two of the richest areas of improvement were 46 (90%) participants endorsing increased comfort level in discussing when to terminate resuscitation efforts (p<0.0001) and 43 (84%) reporting increased comfort level in the use of different resuscitation algorithms that ACLS requires (p<0.0001) (Figure 1 and Figure 2).

**Table 3:** ACLS and PALS Simulation Before and After Evaluation Data (2020 and 2022 Combined Data) Statistical analysis done using paired T-tests ACLS: Advanced Cardiac Life Support, DVT: deep vein thrombosis, PE: pulmonary embolism, PEA: pulseless electrical activity, RCPA: rapid cardiopulmonary assessment, SD: standard deviation, V-tach: ventricular tachycardia

I feel comfortable with my ability to: (N=51) (strongly agree = 5, strongly disagree = 1)	Prior to training			Immediately after training			Comparing prior and after			Percentage improved comfort
	Mean	SD	Median	Mean	SD	Median	Mean difference	p-value	t-value	
Perform complete RCPA	3.96	0.65	4	4.59	0.53	5	0.63	<0.001	7.09	55%
Recognize signs of narcotic overdose and use of reversal agents	3.72	0.62	4	4.49	0.53	5	0.77	<0.001	8.82	67%
Use H’s and T’s mnemonic to diagnose causes of PEA	3.56	0.72	4	4.43	0.60	4	0.87	<0.001	11.63	78%
Use different resuscitation algorithms that ACLS requires	2.70	0.93	3	4.04	0.71	4	1.34	<0.001	11.66	84%
Review the preparation for intubation with SOAPME mnemonic	3.39	0.74	3	4.23	0.70	4	0.84	<0.001	7.94	65%
Discuss when to terminate unsuccessful resuscitation efforts	2.55	1.00	3	4.06	0.75	4	1.51	<0.001	12.93	90%
Recognize torsades de pointes and tailor V-tach resuscitation accordingly	3.24	0.88	3	4.25	0.65	4	1.01	<0.001	9.56	76%
Completing a post-resuscitation stabilization	3.20	0.88	3	4.35	0.48	4	1.15	<0.001	9.64	76%
Discuss risk factors for DVT and PE in children and adolescents	3.65	0.81	4	4.47	0.60	5	0.82	<0.001	8.99	69%
Discuss the common conditions and medications associated with QTc prolongation	3.43	0.85	4	4.27	0.60	4	0.84	<0.001	8.23	67%

Highlights of the free text remarks from the evaluations included the following: “(Some of the most useful aspects of the session were) review of adult-centric resuscitation medications and doses,” “Surprisingly, the team dynamic and collaborative effort still works well in the virtual setting,” and “It was really thought provoking to have the same exact clinical background that led to 3 different (simulation) outcomes.”

**Figure 3:**
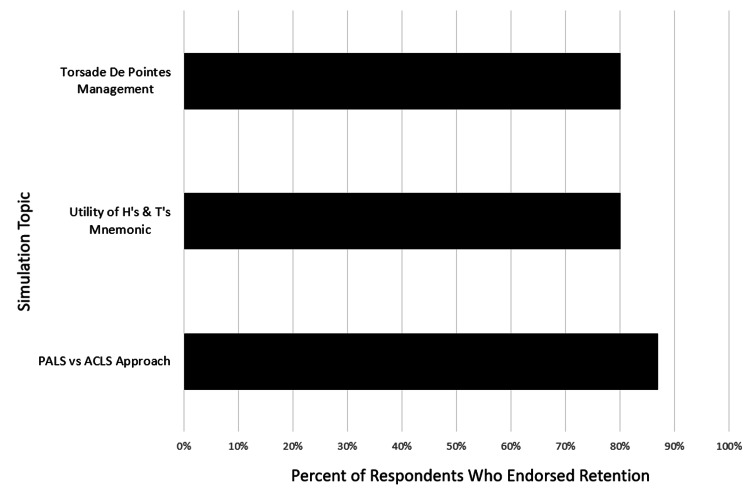
Percent of Supplemental Follow-Up Survey Respondents (n=15) Who Endorsed Retention of Material by Topic ACLS: Advanced Cardiopulmonary Life Support, PALS: Pediatric Advanced Life Support

Supplemental follow-up REDCap survey evaluations, including content recall and use in clinical practice, were completed by 15 participants (seven repeat participants and eight new participants). Results showed that 10 (66%) participants remembered what they learned in the sessions without prompting. When further prompted by details of specific components of the simulation, the number of participants who recalled specifics include the following: 13 (87%) regarding PALS/ACLS approaches, 12 (80%) regarding H’s and T’s, and 12 (80%) related to torsades de pointes management (Figure 3). In addition, free text comments pertaining to participants’ use of the content in their clinical and educational practice noted consistent use of the rapid cardiopulmonary assessment in sick children, consideration of PE and PE risk factors for adolescent patients, and the use of H’s and T’s in diagnostic considerations. One participant noted they had reviewed all the cases with their trainees while on clinical service as a didactic learning opportunity. Another participant noted they had a teenage patient arrest and expire in a community Emergency Department within months of the simulation and felt better prepared to both manage the resuscitation and handle the emotional burden of the case due to the simulation and supplemental debrief session.

Twenty-six participants completed the REDCap 10-question pre- and post-test, where five questions pertaining to simulation content were scored. The results from the pre-test (M=2.9, SD: 0.85) and post-test (M=3.9, SD: 0.81) indicate evidence of knowledge transfer (p<0.001). Overall, 20 (77%) individual respondents had improved post-test scores.

## Discussion

The curriculum content presented here was a natural outgrowth of a well-established simulation curriculum among hospital medicine attendings at a free-standing tertiary children’s hospital. The educators previously clustered content by age (newborn simulation) or organ system (respiratory compromise) and utilized pediatric algorithms exclusively. The novel idea of simulations focused on older pediatric patients skirting the line between pediatric and adult care was one of significant interest. Although literature as early as 2009 wrestles with the dilemma of NRP or PALS when it comes to newborn resuscitation in and out of the delivery room [21-23], the same conversation regarding when to transition resuscitation strategies from PALS to ACLS is lacking in the literature.

Meanwhile, the definition of adolescence can be wide-ranging with some institutions including up to 24 years old [24], and ACLS suggests adult resuscitation methodologies can be instituted after signs of puberty, which can be as early as eight or nine years of age [25]. Many pediatricians practicing inpatient medicine are only required to supplement their basic life support skills with PALS, but the inpatient care of older adolescents and larger adult-sized patients in pediatric hospitals often involves problems seen more frequently in adult populations, such as opioid overdose [26], pulmonary embolism [27], and polypharmacy-induced QTc prolongation [28], which are covered more extensively in ACLS guidelines. As far as we are aware, this is the first study of its kind comparing and utilizing PALS and ACLS principles in medical education.

Additionally, the desire to continue education during the pandemic drove our educators to remote content and development of virtual patient training, with the support of technology and simulation staff for the venture. There is strong evidence that virtual learning, when done correctly, can be an effective and well-received form of education. Synchronous and asynchronous remote learning has been shown to be a reasonable option for adult learners in medicine, with suggested feasibility since 2003 [5-7,12,29]. The breadth of this data has grown significantly since the COVID-19 pandemic, which forced many institutions to implement, and study, remote training and educational programs [11,29,30]. Resuscitation simulation specifically has been shown to be effective and well-received by healthcare professionals in a virtual format [9-11,29]. Notably, however, the majority of this research has been focused on remote-facilitation simulation-based learning [8,11,29], and only a few studies have shown the effectiveness of a fully virtual, animation, and technology-driven program, such as ours [9,10].

Our results indicate that simulation through an electronic remote platform with virtual technology can be successful and well-received with the help of simulation support and appropriate visual aids, which are essential for the needed realism. While critical care experience was necessary for case creation, any skilled simulation facilitator proficient in PALS and ACLS content could run this simulation with preparation. This approach may be useful in resource-limited or geographically dispersed settings, as well as for those who are now investing in remote learning in a post-COVID-19 pandemic environment. We observed that participants found the ACLS and PALS simulation content valuable and educational, with case diagnoses that encouraged learners to think about pediatric and adult treatment guidelines. Increased comfort in providing care for adult patients by primarily pediatric providers has implications beyond the COVID-19 pandemic.

There are several limitations to this work, as well as areas for future evaluation and curriculum development. While this simulation involved over 80 training encounters with physicians working across our hospital system’s various sites, it was led by one facilitator with participants who were a self-selected group of motivated learners. The Likert scale evaluations were captured immediately after simulation completion and did not follow up on perceptions, although 15 (29%) participants did complete a survey later regarding their subjective knowledge retention and use of skills in clinical practice and trainee education. The low response rate to the follow-up survey (15 (27%) participants) limits the ability to fully capture content retention by participants. Pre- and post-tests were completed by 26 (68%) participants. A more robust completion rate would have better represented our participants.

A strength of this simulation program is that while it stresses a particular cohort of patients (in this case, older pediatric and/or adult patients), it relies on the core principles of the rapid cardiopulmonary assessment and the H’s and T’s as diagnostic aids, which are applicable for patients of all ages. These basic principles are applied and reinforced throughout each case. Remote sessions allow for more participation in simulation exercises, as scheduling restraints or geographic distance may limit providers’ ability to participate in person. In addition, our simulations allowed participation by physicians with varying degrees of patient interaction and responsibilities from supervising residents on acute care floors at a tertiary free-standing children’s hospital to covering level 2 neonatal intensive care units (NICUs) and emergency department consults at community hospital sites. Overall learner growth and feedback across this spectrum implies the content is both appropriate for and useful to this wide spectrum of learners. Moreover, the content of the simulation sessions may be applicable to medical learners of all levels working in a variety of settings, although more research would be needed to investigate this possibility.

## Conclusions

Interactive tele-simulation using an online video platform enhanced with visual aids is a useful option when in-person resuscitation simulation is not available. Although hands-on skills are not possible, the visual aids and cases appear to help with suspension of disbelief and clinical decision-making, and the chat function allows for teamwork. Despite older adolescents and obese children being more likely to have common adult conditions such as pulmonary embolism and polypharmacy-induced QTc prolongation, the literature is lacking in research and medical education describing the intersection of PALS and ACLS, making our curriculum the first of its kind. Practice and reinforcement of ACLS algorithms, H’s and T’s, and the rapid cardiopulmonary assessment in pediatrics was found to be meaningful to clinicians who take care of patients of varying ages and sizes. This approach resulted in improved clinician comfort and clinical knowledge with unfamiliar adult-centric scenarios.
